# The intrinsic resistome of bacterial pathogens

**DOI:** 10.3389/fmicb.2013.00103

**Published:** 2013-04-30

**Authors:** Jorge Olivares, Alejandra Bernardini, Guillermo Garcia-Leon, Fernando Corona, Maria B. Sanchez, Jose L. Martinez

**Affiliations:** Centro Nacional de Biotecnología, Consejo Superior de Investigaciones CientíficasMadrid, Spain

**Keywords:** intrinsic resistance, MDR efflux pump, biofilms, swarming, phenotypic resistance, persistence

## Abstract

Intrinsically resistant bacteria have emerged as a relevant health problem in the last years. Those bacterial species, several of them with an environmental origin, present naturally low-level susceptibility to several drugs. It has been proposed that intrinsic resistance is mainly the consequence of the impermeability of cellular envelopes, the activity of multidrug efflux pumps or the lack of appropriate targets for a given family of drugs. However, recently published articles indicate that the characteristic phenotype of susceptibility to antibiotics of a given bacterial species depends on the concerted activity of several elements, what has been named as intrinsic resistome. These determinants comprise not just classical resistance genes. Other elements, several of them involved in basic bacterial metabolic processes, are of relevance for the intrinsic resistance of bacterial pathogens. In the present review we analyze recent publications on the intrinsic resistomes of *Escherichia coli* and *Pseudomonas aeruginosa*. We present as well information on the role that global regulators of bacterial metabolism, as Crc from *P. aeruginosa*, may have on modulating bacterial susceptibility to antibiotics. Finally, we discuss the possibility of searching inhibitors of the intrinsic resistome in the aim of improving the activity of drugs currently in use for clinical practice.

Clinical definition of antibiotic resistance is mainly based on the bacterial response to treatment. One microorganism is considered as resistant if there exists a high likelihood of therapeutic failure upon antibiotic treatment. Consequently, resistance has been operationally defined in the basis of breakpoints of the minimal inhibitory concentrations (MICs). More recently, an ecological definition of antibiotic resistance is emerging, which is based in the MIC value identifying the upper limit of the wild-type population (Turnidge et al., 2006). This is defined as the ecological cut off (ECOFF) value and all strains presenting MICs above ECOFF are considered resistant from an ecological point of view, even if they are classified as susceptible in the basis of clinical breakpoints. When defining the intrinsic resistome, we are facing the same situation. Bacteria are classically considered intrinsically resistant in the basis of the clinical definition, in other words, if their infections cannot be treated with a given antibiotic. Three are the most relevant causes of this intrinsic resistance: lack of the target, activity of chromosomally encoded antibiotic-inactivating enzymes and reduced uptake of the antibiotic, the later includes reduced permeability of the cellular envelopes and activity of efflux pumps (Nikaido, 1989, 1994; Li et al., 1994; Fernandez and Hancock, 2012). More recently, in the same line of the ecological definition of resistance, the “intrinsic resistome” has been defined as the set of elements that contributes directly or indirectly to antibiotic resistance, and whose presence is independent of previous antibiotic exposure and is not due to horizontal gene transfer (HGT; Fajardo et al., 2008; Wright, 2010).

This definition encompasses all the chromosomally encoded elements that have not been recently acquired as the consequence of the recent human use of antibiotics for therapy and farming purposes. Consequently, for any bacterial species an intrinsic resistome can be defined, irrespective on whether or not this species is classified as intrinsically resistant in the basis of clinical breakpoints. From the studies on the intrinsic resistome, two categories of genes have emerged (Fajardo et al., 2008). Those which inactivation make bacteria more resistant to antibiotics and those which inactivation make bacteria more susceptible. The first ones define elements that are relevant for the acquisition of resistance. For instance, the mutation in a transcriptional repressor of a multidrug (MDR) efflux pump turns the microorganism more resistant to antibiotics (Alonso and Martinez, 1997, 2000, 2001). Mapping these elements is important to define the capability of an organism to evolve toward resistance by mutation (Martinez and Baquero, 2000) and is thus relevant for predicting the evolution of resistance (Martinez et al., 2007, 2011a). The other group of elements define the determinants that contribute to the natural phenotype of susceptibility to antibiotics of a given species, and constitute the *bona fide* intrinsic resistome. Inactivation of these elements make bacteria more susceptible to antibiotics, which may be useful for improving efficacy of current drugs (Martinez, 2012). This has been the situation of inhibitors of plasmid-encoded β-lactamases, which have been demonstrated to be efficient drugs to be used in combination with β-lactams (Reading and Cole, 1977). Similarly, the inhibition of a MDR efflux pump (or another mechanism of intrinsic resistance) might also improve the efficacy of antibiotics currently in use or allow the utilization of others (Lomovskaya et al., 1999; Renau et al., 1999; Lomovskaya and Watkins, 2001). For instance, macrolides are not used for treatment of Gram-negative infections because these organisms are intrinsically resistant to this family of antibiotics. However, the major *Escherichia* *coli* efflux pump AcrAB extrudes macrolides and its inactivation might increase the susceptibility of *Escherichia coli* to these antibiotics (Chollet et al., 2004). This evidence indicates that macrolides might be useful for treating Gram-negative infections if they are used in combination with an inhibitor of MDR efflux pumps.

As stated above, the main causes of intrinsic resistance from a clinical viewpoint are lack of the target and the inactivation, low uptake and efflux of the antibiotic. However, all bacterial species harbor in their genomes genes encoding MDR efflux pumps, and several present also chromosomally encoded antibiotic-inactivating enzymes, even though they are not classified as intrinsically resistant from the clinical point of view (Saier et al., 1998; Webber and Piddock, 2003; Piddock, 2006; Poole, 2007; Vila and Martinez, 2008; Nikaido, 2009). The study of the intrinsic resistome of bacterial pathogens has shown that in addition to these elements, several others contribute to the phenotype of resistance. Among them, there are the aforementioned classical resistance genes, but there exist also several other elements belonging to all functional categories, including elements of the bacterial general metabolism (Fajardo et al., 2008). These results indicate that the specific phenotype of susceptibility to antibiotics of a given bacterial species is an emergent property consequence of the concerted action of several elements (Girgis et al., 2009). The large functional diversity of the elements of the intrinsic resistome indicates this has not evolved to specifically counteract the activity of the antibiotics. Together with the proposal that antibiotics might be molecular signals at the low concentrations they are likely present in natural ecosystems (Davies, 2006; Linares et al., 2006; Yim et al., 2006, 2007; Fajardo and Martinez, 2008), this situation allows a complementary view to the traditional weapons/shields roles that antibiotics and their resistance genes may have at natural ecosystems (Martinez, 2008; Aminov, 2009, 2010; Fajardo et al., 2009; Martinez et al., 2009a; Allen et al., 2010; Davies and Davies, 2010; Sengupta et al., 2013).

Studying the intrinsic resistome is of relevance for predicting evolution of resistance (Martinez et al., 2007, 2011a), for understanding the linkage between resistance and other bacterial processes as virulence (Martinez and Baquero, 2002; Martinez et al., 2009a,b) or metabolism (Martinez and Rojo, 2011), and for defining novel targets which inactivation make bacteria more susceptible to antibiotics (Martinez et al., 2011b; Martinez, 2012). In this article we present information of those organisms (*Escherichia coli* and *Pseudomonas aeruginosa*) for which more information on their intrinsic resistome is available. We discuss as well some issues concerning transient phenotypic resistance, which also depends on the intrinsic capabilities of the bacteria for evading antibiotics action (**Figure 1**). Finally, we present updated information on studies on the development of inhibitors of resistance.

**FIGURE 1:**
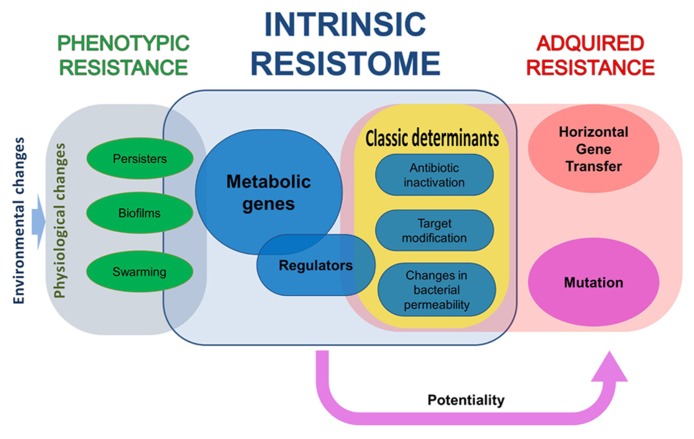
**The different elements in bacterial resistance to antibiotics.** All bacteria have a repertoire of elements that contribute to their characteristic phenotype of susceptibility to antibiotics, what has been dubbed as intrinsic resistome. Some of the elements of this resistome are classical resistance elements, as antibiotic-inactivating enzymes, whereas others belong to all functional categories. The mutation of some of these elements makes bacteria more susceptible to antibiotics, whereas for some others increased resistance is acquired. Nevertheless, acquisition of a phenotype of increased resistance to antibiotics not always implies a genetic change, either because of mutation or as the consequence of the acquisition of a resistance gene by horizontal gene transfer. Phenotypic, non-inheritable resistance can be achieved by different processes that include, among others, growth in biofilms, swarming adaptation, and development of persistence.

## METHODS FOR ANALYZING THE INTRINSIC RESISTOME

Genome-wide analysis of the intrinsic resistome of a given microorganism requires using high-throughput technologies. Among them, the use of insertion or deletion libraries, allows determining the contribution of each single gene to the characteristic phenotype of a given bacteria (Liu et al., 2010). This method is the best suited to determine if the inactivation of a gene changes bacterial susceptibility. However, it does not allow analyzing the effect of mutations that do not fully inactivate a given determinant but changes its activity. Alternatively, the use of plasmid libraries containing each open reading frame of a given genome allows to establish the contribution to resistance of each gene when is overexpressed or when it is transferred to a heterologous host (Soo et al., 2011; Spindler et al., 2013). This method is only useful for analyzing acquired resistance, but not for studying genes which inactivation alters the bacterial susceptibility to antimicrobials. The use of high-throughput sequencing and microarray technologies is also useful for studying intrinsic resistance. These methodologies can be used for comparing populations grown in the absence and in the presence of antibiotics. These populations can be formed by the type of libraries above mentioned (transposon-tagged of expression libraries), in which case the enrichment of mutants or clones in the presence of antibiotics allow defining genes contributing to resistance (Girgis et al., 2009; Zhang et al., 2012; Spindler et al., 2013). While these methods are faster and cheaper than methods based in analyzing the susceptibility of each single mutant/clone, their main drawback is that among all potential determinants that contribute to resistance, only those with lower fitness costs will be enriched and several of the elements that may be detected by classical susceptibility tests would not be detected using enrichment-based technologies. This drawback may be a benefit when the analysis is not made on a library, but using a wild-type strain. In these circumstances, sequencing the evolving population allows in a single step defining the mutations that produce resistance and present the smallest fitness costs among those analyzed, as well as to determine potential compensatory mutations (Gullberg et al., 2011; Toprak et al., 2012). These mutations are the most likely found in clinical isolates (Martinez et al., 2007, 2011a; Shcherbakov et al., 2010).

## THE *Escherichia coli* INTRINSIC RESISTOME

Although *Escherichia coli* is traditionally considered a susceptible organism, acquired resistance to antibiotics was first detected in enteric bacteria, *Escherichia coli*, *Shigella*, and *Salmonella*, in the late 1950s to early 1960s (Watanabe, 1963). Nowadays*, Escherichia coli* accounts for 17.3% of clinical infections requiring hospitalization and is the second most common cause of infection behind *Staphylococcus aureus* (18.8%). Among outpatient infections, *Escherichia coli* is the most common organism (38.6%).

The antibiotic intrinsic resistome of *Escherichia coli* has been studied by testing the susceptibility to several antibiotics of mutants from gene knockout collections (Tamae et al., 2008; Liu et al., 2010) or transposon-tagged mutant libraries (Girgis et al., 2009). The results from these screenings showed that several genes participate in the phenotype of susceptibility to antibiotics in this species. Among them, some are classical resistance genes. Indeed, this bacterium has different known resistance mechanisms; such as the AmpC β-lactamase and MDR efflux systems like AcrAB–TolC (Lindberg and Normark, 1986; Ma et al., 1993; Lubelski et al., 2007; Jacoby, 2009). In addition, *Escherichia coli* harbors several genes that might be of relevance for its susceptibility to antibiotics as those involved in the repair of DNA damage or cell membrane synthesis and integrity. In addition to these comprehensive analysis, other studies based on the whole-genome sequence of strains evolving in the presence of antibiotics (Toprak et al., 2012) or on the enrichment of specific mutants of transposon-tagged libraries grown with antibiotics have been used to track genes relevant for the development of resistance (Zhang et al., 2012). However, in this type of analysis, mutants are competing one to each other and only those with the highest fitness will be selected.

*Escherichia coli* as a Gram-negative bacterium presents two cell membranes, and both are non-specific barriers preventing drug influx into the cell. The lipopolysaccharide (LPS) of the outer membrane protects the cell against hydrophobic antibiotics and polycationic compounds such as aminoglycosides and polymyxins. LPS presents anionic groups and it is the first barrier where some antibiotics bind (Hancock, 1984). Because of this, changes in the LPS can alter the susceptibility to antibiotics. For instance, the loss of *rffA*, a gene encoding an enzyme implicated in the LPS synthesis, increases susceptibility to gentamicin (Tamae et al., 2008). The *Enterobacteriaceae *common antigen (ECA) is also an intrinsic resistance element that provides protection against organic acids (Barua et al., 2002). In a similar way as happens with the LPS, changes in ECA may alter the susceptibility to antibiotics. In line with this reasoning is the finding that inactivation of the ECA biosynthesis protein WzxE increases *Escherichia coli* susceptibility to nalidixic acid and amikacin (Girgis et al., 2009). Mechanisms that control the negative charge of the outer membrane, as the Yrb system and the Pmr regulon, modulate the bacterial susceptibility to neutral or negatively charged compounds, such as nalidixic acid, lomefloxacin, and doxycycline (Girgis et al., 2009).

The gates of entry of several nutrients through the outer membrane are the porins, which are also used by antibiotics for entering into the cell (Nikaido, 1994). Many *Escherichia coli* strains have developed resistance to antibiotics as β-lactams and quinolones by mutations in the genes that encode porins or regulate their expression (Hirai et al., 1986; Adler et al., 2013). In addition, transient down-regulation of the expression of the porins can also trigger phenotypic resistance (Fernandez and Hancock, 2012). A general mechanism that prevents the cellular accumulation of drugs is their active extrusion from the cell or from the cytoplasmic membrane through MDR efflux pumps (Nikaido, 1998a, b). The AcrAB–TolC system of *Escherichia coli* is one of the best-characterized MDR transporters (Ma et al., 1995). This system consists of AcrA, a membrane fusion protein; AcrB, a transporter of the RND family; and TolC, an outer membrane protein. AcrAB–TolC is a major determinant for drug intrinsic resistance in *Escherichia coli* (Sulavik et al., 2001). Furthermore, its expression is increased in the presence of bile salts, which are present in the habitat of *Escherichia coli*, the gut (Thanassi et al., 1997; Rosenberg et al., 2003). This means that *Escherichia coli* might present a lower susceptibility to antibiotics when is growing inside its host as the consequence of the overexpression of the MDR efflux pump AcrAB–TolC. Several other MDR transporters provide resistance to a narrow range of compounds. *Escherichia coli* contains five putative ABC-type MDR-like transporters. However, none of these systems provides an appreciable drug resistance to *Escherichia coli*, excepting YbjYZ (macrolide-specific ABC-type efflux carrier), which confers resistance to macrolides composed of 14- and 15-membered lactones, as erythromycin (Kobayashi et al., 2001; Nishino and Yamaguchi, 2001). The EmrAB is another MDR pump that protects the cell from several chemically unrelated antimicrobial agents, e.g., the protonophores carbonyl cyanide *m*-chlorophenylhydrazone and tetrachlorosalicyl anilide and the antibiotics nalidixic acid and thiolactomycin (Lomovskaya et al., 1995).

In addition to MDR efflux pumps, *Escherichia coli* harbors antibiotic-inactivating enzymes. It is worth mentioning that whilst MDR efflux pumps usually confer low-level resistance to a wide range of compounds, antibiotic-inactivating enzymes confer frequently high-level resistance to antibiotics belonging to a single structural family. All *Escherichia coli* strains have the chromosomally encoded β-lactamase AmpC. Although it contributes to resistance to antibiotics, it is difficult to assume that this is its original functional role if we take into consideration that the natural habitat of *Escherichia coli* is the gut, which microbiota does not include β-lactam producers. AmpC β-lactamases hydrolyze broad and extended-spectrum cephalosporins but are not inhibited by β-lactamases inhibitors such as clavulanic acid (Jacoby, 2009). Considering that β-lactams inhibit peptidoglycan transpeptidation, determinants involved in this process might be important for the susceptibility to these antibiotics. This is the case for *mltB* and *slt*, which encode membrane-bound lytic murein transglycosylases (von Rechenberg et al., 1996) and of *ampG*, which encodes a transporter involved in the recycling of murein (Jacobs et al., 1994). These genes are not only relevant for β-lactam susceptibility. They are also important for the regular physiology of *Escherichia coli*, since the loss of *mltB*, *slt*, *ampG*, and *ampC* is deleterious (Girgis et al., 2009).

Among those determinants involved in the intrinsic resistance of *Escherichia coli*, some deal with the general response to stress, including the repair of damaged DNA. It is known that quinolones produce DNA damage and induce the SOS repair system (Howard et al., 1993). However, other antibiotics with a different mechanism of action as nitrofurantoin and metronidazol can also damage DNA, and the RecF pathway of DNA repair of single-strand breaks and gaps (*recF*, *recO*, *recR*, *recQ*, and *recJ*; Amundsen and Smith, 2003) is important to prevent damages caused by these drugs. It has been reported that the generation of hydroxyl radicals leading to double-strands breaks contributes to cell death caused by bactericidal antibiotics (Kohanski et al., 2008). In this sense DNA repair mechanisms involved in the maintenance of DNA integrity as the RecBCD system or genes involved in the response of the cell to damaging agents (*dinG*, *xseA, xseB*, and *gshB*) constitute a valuable battery of defense mechanisms against antibiotics (Liu et al., 2010). A similar response to stress might be in the basis of the role of ribosomal proteins on intrinsic resistance. Indeed, several genes that encode ribosomal proteins (*rplA*, *rpmE*, *rpmJ*) or proteins involved in their modification (*rimK*) have a role in the susceptibility to a set of antibiotics belonging to different structural families (Liu et al., 2010).

Resistance is also provided by some genes that encode elements that are involved in the regulation of gene expression (*dksA*, *fur*, *hfq*, *hns*, *mfd*, *nusB*, *rseA*, *xapR*, *yciT*; Liu et al., 2010). These genes act in different ways, some of them involved in the stress response. For instance the DksA transcription factor regulates genes involved in double-strand break repair (Meddows et al., 2005). However, other determinants are global regulators of basic processes of the bacterial physiology. This is the case of Fur, that is the master regulator in the response to iron availability (Bagg and Neilands, 1987), an environmental cue with relevance for infection (de Lorenzo and Martinez, 1988; Martinez et al., 1990). This indicates that bacterial physiology and the bacterial response to environmental inputs are cornerstones for the phenotype of susceptibility to antibiotics. In addition to genes with known functions, there are several other determinants of the *Escherichia coli* intrinsic resistome which functional role is not known. This is the case of *yecR* and *yfgC*. Removal of *yfgC* decreases the MIC of vancomycin, rifampicin, and ampicillin (Tamae et al., 2008). *yfgC* has homology to peptidases, and apparently it is located in the bacterial inner membrane (Gardy et al., 2005). The existence of elements of unknown function conferring resistance to several antibiotics bear witness regarding the complexity and the variety of intrinsic resistance mechanisms encoded in the *Escherichia coli* genome.

## THE INTRINSIC RESISTOME OF *Pseudomonas aeruginosa*

*Pseudomonas aeruginosa* is one of the most metabolically versatile bacteria described so far (Lister et al., 2009). This particular feature allows it to colonize multiple environments, being isolated from seawater (Levin and Cabelli, 1972), soil (Green et al., 1974), interacting mutualistically with plant roots (Green et al., 1974; Walker et al., 2004), as a plant pathogen (Walker et al., 2004; Records, 2011) and infecting animals (Petersen et al., 2002), including humans (Stover et al., 2000). In addition of its role in infections, *P. aeruginosa* has been isolated at hospitals in different inorganic surfaces, from ventilation and intubation equipments, contact lens and even in hydrotherapy pools (Morales et al., 2012). This versatility and ability to survive on minimum nutritional requirements (Favero et al., 1971) have made this bacterium one of the most successful nosocomial opportunistic pathogens (Lister et al., 2009).

Not only is this ability to survive anywhere the cause of the ecological success of this organism. This bacterium also shows high levels of antibiotic resistance which often makes impossible its eradication. Evolutionary forces have built its high resistance, and countless elements contribute to intrinsic and acquired resistance of this bacterium, considered a model organism to study mechanisms of resistance (Poole et al., 1993).

Although the recent acquisition of antibiotic resistance genes has been extensively described in *P. aeruginosa* (Woodford et al., 2011), these acquired elements are not the unique cause of antibiotic resistance in* P. aeruginosa*; multiple elements contribute substantially to the resistance of this bacterium. Many works have been performed in order to unravel the intrinsic resistome of *P. aeruginosa. *Transposon-tagged libraries have demonstrated that the inactivation of several genes cause changes in antibiotic susceptibility. The majority of these genes are not related with the cell envelope or efflux pumps and many of them are involved in bacterial metabolism (Breidenstein et al., 2008; Fajardo et al., 2008; Schurek et al., 2008; Alvarez-Ortega et al., 2010; Fernandez et al., 2013). Mutants with changes in susceptibility to ciprofloxacin, aminoglycosides, β-lactams, and polymyxin B have been identified using this method.

Ciprofloxacin is a broad-spectrum fluoroquinolone that target the bacterial enzymes DNA gyrase and topoisomerase IV (Drlica and Zhao, 1997). Thirty-five and 79 mutants with increased and decreased susceptibilities to this antibiotic, respectively (Breidenstein et al., 2008), were identified in a screening of a PA14 mutant transposon library (Liberati et al., 2006). The majority of these mutants demonstrated only twofold changes in susceptibility compared with the respective isogenic strain; just the mutant in *ftsK* was eightfold more susceptible. Mutants in four genes involved in DNA replication and repair (Holliday junction helicase *ruvA*, the ATP-dependent RNA helicase *recG*, recombinase *xerD*, and the site specific recombinase *sss*) present increased susceptibility to ciprofloxacin. Additionally, *ruvA*, *xerD*, and *fstK* grow slower compared with the wild-type. On the contrary, mutants in genes *nuoBDGHIJLN* that encode a dehydrogenase are less susceptible to ciprofloxacin. These mutants in the *nuo* operon present decreased susceptibility to tobramycin also (Schurek et al., 2008) supporting the idea that some of the elements involved in intrinsic resistance have not evolved to counteract the activity of a specific antibiotic (Fajardo et al., 2008; Martinez and Rojo, 2011).

Despite this apparent lack of specificity, there is not a common response to all antibiotics, even for those belonging to the same structural family. In a screening using imipenem, meropenem and ceftazidime just one mutant (PA0908) presented reduced susceptibility to all three antibiotics and two (*glnK* and *ftsK)* showed increased susceptibility to all three antibiotics (Alvarez-Ortega et al., 2010). Even more, the mutant in *ftsK*, which encodes a protein involved in cell division (Lewenza et al., 2005), presents an increased susceptibility to ciprofloxacin too (Breidenstein et al., 2008). In the same way, mutants in many genes that were more susceptible to β-lactams (PA0401, *pyrB* and *pyrD*; Alvarez-Ortega et al., 2010) presented decreased susceptibility to polymyxin B (Fernandez et al., 2013). However, this is not a general trend, since mutants in *galU*, *ampR*, *lptc*, *aroB*, *wapR*, and *ssg *showed decreased susceptibility to β-lactams and they are more susceptible to polymyxin B also (Fernandez et al., 2013). The majority of these genes participate actively in the central metabolism of *P. aeruginosa* (Stover et al., 2000) reinforcing the idea that genes involved in metabolism can play an important role in the intrinsic resistance to antibiotics of the microorganisms.

The cell membrane is considered one of the principal contributors to intrinsic resistance (Nikaido, 1989). In this way mutations in many genes that take part in the LPS synthesis in *P. aeruginosa* produce changes of the susceptibility against β-lactams and tobramycin. A mutant in *wapR*, which encodes a protein involved in the biosynthesis of the LPS core (Poon et al., 2008), is less susceptible to ceftazidime and meropenem; mutations in adjacent genes (PA5001, PA5002, PA5003, and PA5005) which participate in the LPS synthesis also present reduced susceptibility to ceftazidime and some mutants present cross resistance with meropenem (Alvarez-Ortega et al., 2010). A similar effect was observed in mutants of genes involved in the O-antigen synthesis (*wbpZ, wbpY, wzt, wzm*, and *wbpW*). These mutants presented decreased susceptibility to tobramycin, demonstrating the importance of the cellular membrane as a barrier to avoid the entrance of antibiotics inside the cell (Schurek et al., 2008).

Efflux pumps contribute considerably to antibiotic resistance in *P. aeruginosa.* They are involved in the extrusion of toxic substances, including antibacterial compounds, from inside the cell to the external environment (Webber and Piddock, 2003). In this bacterium, 12 different RND-type efflux systems (Blair and Piddock, 2009) that can eventually contribute to antibiotic resistance have been described. However, only MexAB-OprM has shown to play a relevant role on the intrinsic resistance of *P. aeruginosa* to antibiotics (Kohler et al., 1997). Expression of the other efflux pumps under standard growing conditions is too low to achieve resistance, an issue that is also frequently described for other bacteria (Grkovic et al., 2001, 2002). However, MDR efflux pumps can be overexpressed, and hence render resistance, either because of mutations in their regulatory elements or because of the presence of effectors that trigger their expression (Grkovic et al., 2001, 2002; Blair and Piddock, 2009; Hernandez et al., 2009, 2011).

Since intrinsic resistance is a multifactorial phenomenon, it might be modulated by global regulators. This is the case of the *P. aeruginosa* “catabolite repression control” regulator Crc (MacGregor et al., 1991). Crc is a post-transcriptional repressor that regulates the use of preferred carbon sources in nutrient-complex ecosystems (Morales et al., 2004; Moreno et al., 2009). Recent work has shown that inhibition of Crc makes *P. aeruginosa* less virulent and more susceptible to different antibiotics (Linares et al., 2010), the latter being a consequence of increased expression of transporters and changes in the LPS composition. This shows the existence of networks connecting antibiotic resistance, bacterial virulence and metabolism. The hubs of these networks may be good targets in the search of novel antimicrobials targeting simultaneously resistance, virulence, and bacterial physiology.

## PHENOTYPIC RESISTANCE

Most studies on antibiotic resistance are based in the analysis of resistant organisms that have acquired the phenotype of resistance as the consequence of genetic, inheritable, changes, which can be mutations (including gene rearrangements) of the acquisition, through HGT, of resistance genes (Walsh, 2000). However, there are other situations, grouped under the name of phenotypic resistance, in which bacteria present a non-inheritable situation of resistance to the antibiotics (Levin, 2004; Levin and Rozen, 2006). Phenotypic resistance is thus defined as a transient situation in which a bacterial population, usually susceptible to antibiotics, is transiently resistant (**Figure 1**). The elements contributing to this phenotype are a part of the intrinsic resistome that are only unveiled under specific growing conditions (Martinez et al., 1994). Below, some examples of phenotypic resistance are described.

### PERSISTENCE

Persistence is defined as a situation in which a bacterial subpopulation is not killed by a given antibiotic under conditions in which the bulk of the population is inhibited. Once antibiotic is removed and growth resumes, persistent cells behave as antibiotic susceptible as the original population, which means that persistence is not the consequence of a genetic change. The existence of persister cells into a population is known since 1944, when this phenomenon was first reported for staphylococcal infections treated with penicillin (Bigger, 1944). Currently, persistence has been described for many other bacterial species and antibiotics. As stated, persisters are phenotypic variants that present increased resistance to antibiotics and are genetically identical to the wild-type. The percentage of persisters in a given culture can be as high as 1% of stationary-phase cells for several microorganisms (Kim and Wood, 2010). Establishment of a persistent subpopulation does not require previous antibiotic exposure.**

The presence of persister cells in infections increase the chance of survival of the bacteria in the presence of antibiotics, an issue particularly relevant in the case of chronic infections. Because of this, the analysis of the genes and mechanisms responsible for the development of a persister phenotype is of relevance to implement novel therapeutic approaches based on the eradication of antibiotic resistant persistent cells.

The first gene described to be involved in the development of persistence was *hipA *(Moyed and Bertrand, 1983) in *Escherichia coli*. HipA is a toxin that belongs to the *hipBA* toxin/antitoxin system (TA systems; Correia et al., 2006), which overexpression inhibits cell growth and induces antibiotic resistance by persister formation. Other TA systems as MqsRA (Kim and Wood, 2010) or TisAB (Dorr et al., 2010; Lewis, 2010) have been also associated to the formation of persister cells, indicating that a misbalance in the production of toxin and antitoxin may be in the basis of this phenotype.

Toxin/antitoxin systems are highly distributed among different bacterial species. Usually, these systems are formed by two components, a toxin that inhibits cell growth and an antitoxin that impedes the activity of the toxin. At the moment, three general TA systems have been described. The antitoxins of TA types I and III are small RNAs, whereas the toxins of the type II TA systems are inhibited by protein antitoxins (Gerdes et al., 1997, 2005; Blower et al., 2011). Recent work has shown that type II TA systems are highly relevant for developing persistence in *Escherichia coli* and that the simultaneous deletion of 10 TA loci from the chromosome of *Escherichia coli* reduced the fraction of persistent cells by at least 100-fold (Maisonneuve et al., 2011; Gerdes and Maisonneuve, 2012).

In addition to TA systems, other factors modulate the development of persistence. Among them, elements involved in the regular bacterial metabolism have shown to be relevant. This is the case of *phoU*, which encodes a negative global regulator that suppresses the cellular metabolic activity, altering expression of several elements with relevance for bacterial physiology, ranking from flagella-encoding genes to genes encoding energy production enzymes (Li and Zhang, 2007).

In depth analysis on the mechanisms of persistence has shown that there are two types of persistent cells (Balaban et al., 2004), which further supports the idea that there exist different routes toward the development of persistence. Type I persisters constitute a pre-existing population of resting cells that are generated at stationary phase. These cells when are inoculated into fresh medium from stationary phase switch back to growing cells with a characteristic extended time lag. An example of genes involved in this phenotype is *hipA7* (Moyed and Bertrand, 1983). Type II persisters constitute a subpopulation of slowly growing cells. An example of genes involved in this phenotype is *hipQ* (Wolfson et al., 1990).

In agreement with the potential role that both bacterial metabolism and TA systems may have on the establishment of persistence, transcriptomic analysis of persister cells have shown that persistence is associated with the down-regulation of biosynthetic genes and the up-regulation of several TA modules (RelBE, MazEF, DinJYafQ, YgiU; Lewis, 2010), Some of these TA systems are known to affect translation (Christensen et al., 2001; Pedersen et al., 2002), which explains their effect in dormancy and consequently on antibiotic resistance. The SOS response, which up-regulates DNA repair functions, also induces several TA genes in *Escherichia coli*, whose promoters contain a Lex box: *symER, hokE, yafN/yafO*, and *tisAB/istR* (Dorr et al., 2010). This means that TA systems, and hence the formation of persister cells, can be activated by factors that trigger the SOS system. It is known that some antibiotics, such as quinolones, induce the emergence of persister cells (Dorr et al., 2010). Since these antibiotics produce DNA damage and activate the SOS system, it is likely possible that the effect of quinolones on persistence is SOS-dependent. The role of other stresses on persistence has been studied. This is the case of oxidative stress that is important during infection due to the immune response. However, this stress only induces persistence against fluoroquinolones but not for other antibiotics such as kanamycin. Surprisingly, this phenotype is due to the overexpression of the MDR efflux pump AcrAB–TolC (Wu et al., 2012). This indicates that transient expression of classic antibiotic resistance mechanisms forming part of the intrinsic resistome may be of relevance for the establishment of persistence.

One important issue for avoiding persistence would be finding elements which mutation impede the development of persistent cells. However, the analysis of a transposon mutants library of *Escherichia coli* (Hu and Coates, 2005; Hansen et al., 2008) and of the comprehensive Kei collection (Baba et al., 2006), did not allow identifying any gene which mutation fully impede the phenotype of persistence. These data suggest a great degree of redundancy in the elements and pathways involved in the development of persistence. Even though TA systems could be good candidates to decrease the fraction of persister cells in a given population, their high number, with 671 TA systems already identified in 126 prokaryotic genomes (Pandey and Gerdes, 2005) makes difficult developing drugs directed to reduce persistence by targeting these systems.

Another approach for eliminating persisters can be by shifting their metabolic state. A recent report shows that this is feasible by specific metabolic stimuli that allow the recovery of the proton motive force of persistent cells, enabling their killing by aminoglycosides (Allison et al., 2011).

### BIOFILM

Biofilm is a structured population of bacteria embedded in a matrix, which is composed by polysaccharides, proteins, and extracellular DNA. It has been shown than cells growing in biofilms are less susceptible to antibiotics than those growing planktonically (Mah and O’Toole, 2001; Amini et al., 2011). This phenotypic resistance is relevant for the treatment of infections on intubated or catheterized patients, as well as in infections of prosthesis and some chronic infections as those of cystic fibrosis patients that involve the colonization of surfaces (Costerton et al., 1999) in which biofilm formation is frequent. The antibiotic resistance associated with biofilms depends on several causes, some due to the structure of the extracellular matrix, some other to the physiological state of biofilm-growing bacteria; which is different to that of planktonic cells. Even inside the biofilm, bacteria show different metabolic states, because there is a gradient of nutrients and oxygen between the surface of the biofilm and its deeper region.

The extracellular matrix may change the activity of the antibiotics by two different reasons; by diminishing the diffusion of the antibiotic or by sequestering it through its binding to the matrix. This is not a general trend, since in several occasions, slow diffusion of the antibiotic is not the most important element in the phenotypic resistance displayed by biofilms (Walters et al., 2003; Stewart et al., 2009; Singh et al., 2010). Another aspect in which the extracellular matrix may participate in the phenotype of resistance of biofilms is by triggering specific mechanisms of resistance. DNA, a macromolecule capable of chelate cations, is one of the components of the extracellular matrix. Since a reduced concentrations of divalent cations trigger expression of the regulator of resistance to cationic antimicrobials PhoP–PhoQ (Mulcahy et al., 2008), the extracellular matrix trigger itself resistance to these drugs.

When analyzing the role of the metabolic state of bacteria on the phenotypic resistance of biofilms, it has been shown that the degree of resistance depends on the region of the biofilm and on the antibiotic involved. Different regions of the biofilms contain subpopulations in different metabolic stages that mainly depend on the oxygen and nutrients availability (Huang et al., 1995; Sternberg et al., 1999). It has been described than oxygen-rich regions of *P. aeruginosa* biofilms are highly susceptible to quinolones whilst cells in these regions are phenotypically resistant to cationic peptides, and the opposite occurs at regions of the biofilms with low oxygen tension (Rani et al., 2007). Altogether, these results indicate that the phenotypic resistance of bacterial biofilms depend on several factors that operate simultaneously.

### SWARMING

Swarming is a specific type of movement of bacterial populations. It is characterized by the formation of hyper-flagellated cells in nutrient-rich environments. It is supposed that this type of motility allows the colonization of such environments. Consequently, swarming is just the most visible phenotype of a complex physiological adaptation process that is dependent on cell–cell signaling [quorum-sensing (QS)] and on nutrients availability (Fraser and Hughes, 1999). The capability of swarm has been described in different microorganisms, including *Escherichia coli, Serratia marcescens, Burkholderia thailandensis, Bacillus subtilis*, *Salmonella enterica* serovar Typhimurium, and *P. aeruginosa *(Kim et al., 2003; Overhage et al., 2008a; Lai et al., 2009), and it might be of relevance for the colonization of surfaces during infection, as for instance in the lungs of cystic fibrosis patients. A relevant characteristic of swarmer cells consists on their reduced susceptibility to different antibiotics (Kim et al., 2003; Overhage et al., 2008b; Lai et al., 2009).

Transcriptomic analyses of *P. aeruginosa* have shown that several genes change their expression during swarm cell differentiation. Some of these genes encode porins and efflux pumps, such as MexGHI-OpmD. A differential expression of these genes in swarmer cells might be involved in their phenotype of reduced susceptibility to antibiotics (Overhage et al., 2008a). Nevertheless, the reasons for this transient phenotype of antibiotic resistance are still far to be fully understood, since there are other elements that might be involved in the phenotype. For instance, several proteases (Lon, AsrA, PfpI, ClpS, and ClpP) that affect swarming motility are also relevant for the formation of biofilms, and therefore for phenotypic antibiotic resistance (Marr et al., 2007; Kindrachuk et al., 2011; Fernandez and Hancock, 2012).

Quorum-sensing seems to play a key role in swarming differentiation. It has been shown that PvdQ, an acylase that hydrolyzes the QS signal 3-oxo-C12-HSL [*N*-(3-oxo-dodecanoyl)-l-homoserine lactone] is involved in swarming. The analysis of cells either overexpressing or lacking PvdQ showed that PvdQ reduced *P. aeruginosa *outer membrane permeability, thereby elevating antibiotic resistance under swarming conditions, upon which this protein is up-regulated (Wang et al., 2013). A similar effect of reduced permeability has been shown for *Salmonella enterica* swarmer cells. In particular the expression of the porin OmpA*, *which is used for the entrance of nutrients and some antibiotics is low in swarming cells (Sugawara and Nikaido, 1992; Kim and Surette, 2004), suggesting that changes in the permeability of the cellular envelopes, in response to nutrients’ availability, might be in the basis of the antibiotic resistance phenotype displayed by swarmer cells.

There are some common factors that might induce the different situations of phenotypic resistance above described. Changes in the environment as the lack of nutrients or low oxygen levels, which reduce the growth rates, occur in stationary phase and this induces persisters, which are much more abundant in this growth phase. These nutritional cues also take place in some regions of bacterial biofilms, mainly at their deepest zone, and are relevant for their reduced susceptibility to antibiotics. QS signaling is a relevant system in the basis of biofilm formation, which is also involved in triggering the bacterial physiological reprogramming that occurs in swarmer cells. Expression of TA systems occurs in response to external signaling, and is involved in the generation of persister cells, but also are differentially expressed during biofilm formation, directly or *via* QS signaling. Cellular damage produced by toxic compounds, antibiotics included, may induce the SOS response, which triggers expression of reparation systems and usually also reduces metabolic activity, a situation that induces the formation of persister cells and is also foreseen in biofilms.

It thus seems that bacterial populations have developed a battery of mechanisms to respond to stress and these mechanisms are also useful to transiently resist the activity of antibiotics.

## INHIBITORS OF RESISTANCE DETERMINANTS

One of the areas under development in the search for novel drugs to fight infections is the study of inhibitors of resistance (Baquero et al., 2011; Martinez et al., 2011b; Martinez, 2012). The use of these drugs in combination with those antibiotics against which the mechanisms of resistance operate, increases bacterial susceptibility to the antimicrobials. The first inhibitors of resistance were developed against mechanisms acquired through HGT, in particular plasmid-encoded β-lactamases (Bush, 1988). However, the definition of intrinsic resistome offers the possibility of developing inhibitors that increase susceptibility to all isolates of a given species (Garcia-Leon et al., 2012). For instance, the inhibition of MDR efflux pumps, which makes Gram-negative bacteria susceptible to macrolides, will allow the use of these compounds for treating infections by Gram-negative microorganisms.

Although clavulanic acid, the first inhibitor of β-lactamases was described more than 40 years ago (Reading and Cole, 1977), few other inhibitors, all of them inhibiting the same type of β-lactamases, are already in the market. Main efforts have focused on the inhibitors of other β-lactamases (Drawz and Bonomo, 2010) and MDR efflux pumps (Lomovskaya and Watkins, 2001). In the case of aminoglycoside-inactivating enzymes, the development of inhibitors is problematic due to the large number and variability of families of these resistance elements.

Based on the Ambler’s classification (Ambler et al., 1991), the β-lactamases are divided into four classes: class A, C, and D being serine β-lactamases, and class B being metallo-β-lactamases (MBLs). The commercially available β-lactamase inhibitors (BLIs), clavulanic acid, sulbactam and tazobactam, are only effective against class A β-lactamases. However, their combinations with β-lactam antibiotics are still effective, despite the emergence of resistance, either because of gene-dosage effect (Martinez et al., 1987, 1989; Reguera et al., 1991), either because of mutations that make β-lactamase resilient to inhibition (Blazquez et al., 1993; Canica et al., 1998; Salverda et al., 2010; Frase et al., 2011; Li et al., 2012).

The discovery that carbapenems may inhibit some β-lactamases opened the possibility of finding antibiotics with dual activities, antimicrobial and inhibitor of resistance (Buynak, 2006). However, this can be a difficult task, since antibiotics as cefoxitin may either inhibit or induce expression of β-lactamases depending on their concentration (Tsuey-Ching et al., 2012). Because of this, mathematical models are being implemented to establish reliable dosage regimes (Bhagunde et al., 2012) and novel derivatives are being developed to surpass these problems. Among them, *N*-acyl β-sultams obtained, by sulfonylation of β-lactams and addition of an acyl group, inhibit *Enterobacter cloacae* class C β-lactamase (Page et al., 2003). Penicillin and cephalosporin sulfone derivatives are also part of the pipeline of BLIs. LN-1-255 is a penicillin sulfone that is active against OXA-type β-lactamases and is being used as model for further improvements in this type of inhibitors (Pattanaik et al., 2009; Drawz et al., 2010).

In addition to β-lactams that inhibit β-lactamases, there are also non-β-lactams able to inhibit these enzymes. One of the families receiving more attention is the one formed by boronic acids (Kiener and Waley, 1978), which are potential inhibitors of all types of serine β-lactamases (Tan et al., 2010), and seem to be effective even against penicillin-binding proteins (PBPs) resistant to β-lactams (Zervosen et al., 2012). Nevertheless clinical use of these compounds is compromised because of the toxicity of boron. Another class of non-β-lactam BLIs is formed by the diazabicyclooctanes (DBOs). There are two compounds of this family under clinical trials: MK-7655 and NXL104 (avibactam) (Coleman, 2011). MK-7655 is a potent inhibitor of class A and C β-lactamases, that can be used in combination with imipenem to kill AmpC and KPC (*Klebsiella pneumoniae* carbapenemase) producers (Hirsch et al., 2012). Avibactam is a promising BLI that inhibits class A and C β-lactamases, including BlaC from *Mycobacterium tuberculosis* (Xu et al., 2012). In combination with ceftazidime, avibactam protects β-lactams from hydrolysis in β-lactamase-producing *Enterobacteriaceae* and *P. aeruginosa* (Dubreuil et al., 2012; Levasseur et al., 2012). More recently it has been proposed that a triple combination avibactam, ceftazidime, and metronidazole will be useful for treating complicated intra-abdominal infections, which may present a mixed population of *Enterobacteriaceae *and anaerobes (Dubreuil et al., 2012; Lucasti et al., 2013).

Finding common inhibitors for all MBLs is a difficult task, due to the diversity of class B β-lactamases (Drawz and Bonomo, 2010). However, different structural families of inhibitors are under development, including tetrahydropyrimidine-2-thione and pyrrole derivatives (Hussein et al., 2012), 3-mercapto-1,2,4-triazoles and *N*-acylated thiosemicarbazides (Faridoon et al., 2012), *N*-heterocyclic dicarboxylic acids and pyridylmercaptothiadiazoles (Feng et al., 2012), 2-substituted 4,5-dihydrothiazole-4-carboxylic acids (Chen et al., 2012), or mercaptoacetate (Wachino et al., 2012).

Although most inhibitors of β-lactamases target a specific family of these enzymes, efforts have been made to develop drugs capable to inactivate a large range of β-lactamases. Among them single molecules as mercaptomethylpenicillinates (Buynak et al., 2004) or reverse hydroxamates and oximes (Ganta et al., 2009) are under development as well as combinations of compounds as BAL30376, which is a mixture of BAL19764, a siderophore monobactam active to class B MBLs, BAL29880, a bridged monobactam active to class C β-lactamases, and clavulanate (Livermore et al., 2010; Page et al., 2011).

In addition to traditional methods in searching enzyme’s inhibitors, information derived from other studies may help in developing β-lactamases inhibitors. In this regard, the finding of a small protein, produced by the same producer of clavulanic acid, *Streptomyces clavuligerus* (Reading and Cole, 1977; Yuan et al., 2011) and capable to inhibit class A β-lactamases opens the possibility of developing peptides or haptamers capable to inhibit β-lactamases. Similarly, the finding that the active site structures and the catalytic mechanisms of N-terminal nucleophile hydrolase (a component of the bacterial proteasome) and β-lactamases are similar, allows discovering cross-inhibition of both enzymes by compounds as *O*-aryloxycarbonyl hydroxamates (Pelto and Pratt, 2008) or 1,3,4-oxathiazol-2-ones (Adediran et al., 2012). This opens the possibility of testing already known inhibitors of the bacterial proteasome as inhibitors of β-lactamases.

Beta-lactamases inhibitors will be very useful, but only for treating infections by those organisms presenting a β-lactamase. A wider spectrum of activity might have the efflux pumps inhibitors (EPIs). MDR efflux pumps are present in all bacterial species contributing to intrinsic and acquired (when overexpressed) resistance to all family of drugs. Since any single efflux pump can extrude a wide range of antibiotics belonging to different structural families, its inhibition will simultaneously increase the bacterial susceptibility to several antibiotics (Vila and Martinez, 2008). *In vitro *work has shown that inhibition of efflux pumps makes bacteria more susceptible to antibiotics and also reduces the probability of emergence of antibiotic resistant mutants (Lomovskaya et al., 2001). In addition, since some efflux pumps contribute to the virulence of bacterial pathogens, their inhibition will also impair their capability for producing infections (Hirakata et al., 2009), including the formation of biofilms (Baugh et al., 2012).

Theoretically, there are different alternatives for inhibiting MDR determinants (Fernandez and Hancock, 2012). One that could serve for inhibiting all efflux pumps would be the inhibition of energy sources required for pumps activity. Unfortunately activity of MDR efflux pumps is coupled either to the membrane potential, either to ATP, both of which are key element for any eukaryotic of prokaryotic cell. This means that most of these potential inhibitors will be too toxic to be used. There are, however, some elements that are specific for bacteria and could be used as potential targets. This is the case of TonB, a protein involved in the activity of *P. aeruginosa *MDR efflux pumps by coupling the energized state of the membrane to the operation of bacterial transporters (Zhao et al., 1998).

Other ways of inhibiting the activity of efflux pumps include the interference with the pump assembly or the blockage of its activity, for instance using antibodies as has been described for SmrA from *Stenotrophomonas maltophilia* (Al-Hamad et al., 2011), the development of effectors precluding the release of the MDR repressor from its operator DNA, and the competition with antibiotics transported by the efflux pump.

One of the first EPIs with potential therapeutic use is the synthetic dipeptide amide phenylalanine-arginyl-β-naphthylamide, which inhibits several Gram-negative efflux pumps (Lomovskaya et al., 2001; Mahamoud et al., 2006), although not all of them (Sanchez et al., 2003). This molecule is a competitive inhibitor that binds to the same site used by the pump to bind the antibiotic. Some efforts have been made in the optimization of diamide-containing EPIs (Watkins et al., 2003). However, it has been shown that the moieties that are responsible for their unfavorable toxicological properties, are also essential for their activity, a situation that impedes their therapeutic use (Lomovskaya and Bostian, 2006).

Pyridopyrimidines and arylpiperazines have also been assayed as EPIs of MDR pumps and major efforts have been performed for their optimization (Nakayama et al., 2004a, b; Yoshida et al., 2006a, b, 2007). Differing to the previously described dipeptide amides, that only impede the action of the antibiotics they compete, pyridopyrimidines increase the susceptibility to all substrates of the efflux pumps, indicating a different mechanism of action (Lomovskaya and Bostian, 2006).

Although some efforts on the development of EPIs against efflux pumps for Gram-positive organisms, as *Staphylococcus aureus* NorA, have been made (Markham et al., 1999; Holler et al., 2012b; Sabatini et al., 2012), most EPIs so far described inhibit efflux pumps from Gram-negative organisms with some degree of specificity. Among them, pyridopyrimidines inhibit MexAB-OprM of *P. aeruginosa* (Yoshida et al., 2007), 1-(1-naphthylmethyl)-piperazine reversed multidrug resistance in *A. baumannii *but not in *P. aeruginosa *(Pannek et al., 2006), or 13-cyclopentylthio-5-OH tetracycline (13-CPTC), a semisynthetic tetracycline analogue that binds TetB pump of *Escherichia coli* (Nelson and Levy, 1999).

The finding that plant-produced compounds are substrates and inducers of efflux pumps (Matilla et al., 2007) suggests that these compounds may also inhibit MDR determinants. Indeed, it has been shown that plant extracts contain a variety of EPIs (Tegos et al., 2002; Musumeci et al., 2003; Lewis and Ausubel, 2006; Stavri et al., 2007), which may be useful for increasing the susceptibility to antibiotics of different bacterial species (Groblacher et al., 2012a, b; Holler et al., 2012a; Roy et al., 2012; Zhou et al., 2012).

In addition of inhibitors targeting specifically classical resistance elements, the study of the intrinsic resistome opens the possibility of looking for inhibitors of targets that contribute to resistance despite they are not classical resistance determinants. This is the case of the *P. aeruginosa *cyanide-insensitive terminal oxidase. Mutants defective in this gene are hypersusceptible to antibiotics (Tavankar et al., 2003). Consequently, inhibition of this component of the respiratory chain will increase the overall susceptibility of *P. aeruginosa* to antibiotics. Same situation happens with global regulators as the *P. aeruginosa* Crc post-transcriptional repressor (Morales et al., 2004; Moreno et al., 2009). Mutants defective in this gene are hypersusceptible to different antibiotics (Linares et al., 2010); hence its inhibition will increase susceptibility to these antibiotics.

An interesting approach in the search of inhibitors of resistance is the screening of non-antibiotic compounds, which had been already tested for other diseases, and may be used as helper compounds to improve efficacy of antibiotics (Martins et al., 2008). The benefit of using these compounds is that their use has been already approved, so as novel long and costly toxicological trials are not needed. Among them some anesthetics, antihistaminic, and psychotherapeutic compounds have demonstrated to improve the activity of antibiotics (Kristiansen and Amaral, 1997). Within this group of compounds some of them change the permeability of the bacterial membrane (Martins et al., 2008), as the anti-inflammatory drug diclofenac (Dutta et al., 2007) or the anti-psychotic chlorpromazine (Blair and Piddock, 2009), whereas for others, the mechanism of action is not known.

A more recent approach for improving the activity of the antibiotics is by enhancing the cellular responses associated to the antibiotics induced cell death pathway. It has been proposed that bactericidal antibiotics induce a cell pathway that involves the generation of oxygen reactive species (Kohanski et al., 2007, 2010). Understanding this pathway may reveal targets for adjuvants that improve the efficacy of the antibiotics (Farha and Brown, 2013). By using *Escherichia coli* whole-genome metabolic models and further experimental validation of predicted targets, it has been shown that inactivation of some elements increase susceptibility to oxidants and antibiotics (Brynildsen et al., 2013), which opens the possibility of searching a new family of drugs capable to increase the activity of antibiotics.

## Conflict of Interest Statement

The authors declare that the research was conducted in the absence of any commercial or financial relationships that could be construed as a potential conflict of interest.
